# Optimal Monochromatic Energy Levels in Spectral CT Pulmonary Angiography for the Evaluation of Pulmonary Embolism

**DOI:** 10.1371/journal.pone.0063140

**Published:** 2013-05-07

**Authors:** Jiejun Cheng, Yan Yin, Huawei Wu, Qing Zhang, Jia Hua, Xiaolan Hua, Jianrong Xu

**Affiliations:** Department of Radiology, Renji Hospital, Shanghai Jiao Tong University School of Medicine, Shanghai, People's Republic of China; Mayo Clinic College of Medicine, United States of America

## Abstract

**Background:**

The aim of this study was to determine the optimal monochromatic spectral CT pulmonary angiography (sCTPA) levels to obtain the highest image quality and diagnostic confidence for pulmonary embolism detection.

**Methods:**

The Institutional Review Board of the Shanghai Jiao Tong University School of Medicine approved this study, and written informed consent was obtained from all participating patients. Seventy-two patients with pulmonary embolism were scanned with spectral CT mode in the arterial phase. One hundred and one sets of virtual monochromatic spectral (VMS) images were generated ranging from 40 keV to 140 keV. Image noise, clot diameter and clot to artery contrast-to-noise ratio (CNR) from seven sets of VMS images at selected monochromatic levels in sCTPA were measured and compared. Subjective image quality and diagnostic confidence for these images were also assessed and compared. Data were analyzed by paired *t* test and Wilcoxon rank sum test.

**Results:**

The lowest noise and the highest image quality score for the VMS images were obtained at 65 keV. The VMS images at 65 keV also had the second highest CNR value behind that of 50 keV VMS images. There was no difference in the mean noise and CNR between the 65 keV and 70 keV VMS images. The apparent clot diameter correlated with the keV levels.

**Conclusions:**

The optimal energy level for detecting pulmonary embolism using dual-energy spectral CT pulmonary angiography was 65–70 keV. Virtual monochromatic spectral images at approximately 65–70 keV yielded the lowest image noise, high CNR and highest diagnostic confidence for the detection of pulmonary embolism.

## Introduction

Multidetector computed tomography (MDCT) has become the first-line imaging test for the assessment of patients with suspected acute pulmonary embolism (PE) [1]–[5]. Recent technological advances in MDCT have led to dual-source or single-source energy CT with fast tube voltage switching, which can detect perfusion defects beyond obstructive clots [6]–[15]. Initial studies have focused on testing the diagnostic accuracy of the perfusion map generated by using dual-energy CT. However, we have also shown spectral CT imaging generated both monochromatic CTPA images for morphologic analysis of PE and material decomposition images for quantitative depiction of pulmonary blood flow and perfusion defects with the emphasis on the latter [16].

Several studies have focused on the effective use of monochromatic images from spectral CT scans by selecting the optimal energy level (keV) to improve image quality and diagnostic confidence in the brain [17], liver [18], [19], thoracic aneurysms [20] and pulmonary embolism [21]. These studies focused primarily on the contrast between the vessel and the background tissue [12], [18], [22] rather than testing the endoluminal changes at various peak kiloelectronvolt levels, or the possibility of reducing iodine load at a lower monochromatic energy level [23]. However, there is an inherent limitation with this technique, particularly when attempting to evaluate the blood volume on perfusion maps and the nonvascular structures or non-enhancing endoluminal clots in enhancing vessels.

The purpose of this study was to investigate the optimal monochromatic level in spectral CT pulmonary angiography for detecting and evaluating pulmonary embolism.

## Methods

### Ethics Statement

All research procedures were approved by the Institutional Review Board of the Shanghai Jiao Tong University School of Medicine and were conducted in accordance with the Declaration of Helsinki. Written informed consent was obtained for all patients.

### Patient Population

From November 2010 to June 2012, 72 patients (49 men, 23 women; mean age: 62.1 y) with pulmonary embolism underwent CT pulmonary angiography (CTPA) with spectral CT imaging mode. Underlying cardiopulmonary disease was excluded in all patients by history and CT findings.

### CT Acquisition Protocol

All patients were examined on a Discovery CT750HD (HDCT) scanner (GE Healthcare, Waukesha, WI, USA). A scout scan was taken first to plan for the spiral acquisition, which included the entire chest from the first ribs to the diaphragm. After scout CT scanning, nonenhanced helical scanning was performed in the conventional helical mode at a tube voltage of 120 kVp. Patients were then injected with a total of 80–100 mL (1.35 mL per kilogram of body weight) nonionic iodinated contrast material ([Iopamidol] 370 mg/mL; Shanghai Bracco Sine Pharmaceutical Co., Ltd., China) with a 4.0 mL/s injection rate followed by 50 mL saline solution via a power injector. The contrast-enhanced CT was obtained using spectral imaging mode with fast tube voltage switching between 80 kVp and 140 kVp on adjacent views during a single rotation. The CT scan was triggered by a bolus tracking technique with the region of interest placed in the pulmonary trunk, and image acquisition started 7 s after the signal attenuation reached the predefined threshold of 100 Hounsfield units (HU). The other spectral CT parameters were as follows: 0.6 s tube rotation time, 1.25 mm collimation, 600 mA tube current, 1.375 pitch, 5 mm/5 mm slice thickness and interval for axial images, 500 mm scan field of view (FOV). The rotation time of 0.6 s was shared by alternating the 80 kVp and 140 kVp with uneven distribution to balance the signals. Gemstone Spectral Imaging (GSI) viewer software (GE Healthcare) was used to process projection data and review images. The two sets of high and low energy projections registered in time and space were used for reconstruction with projection-based material decomposition software and the manufacturer's “Standard” reconstruction kernel. One hundred and one sets of VMS images were generated together with the material decomposition image sets.

### Data Analysis

**Figure 1:**
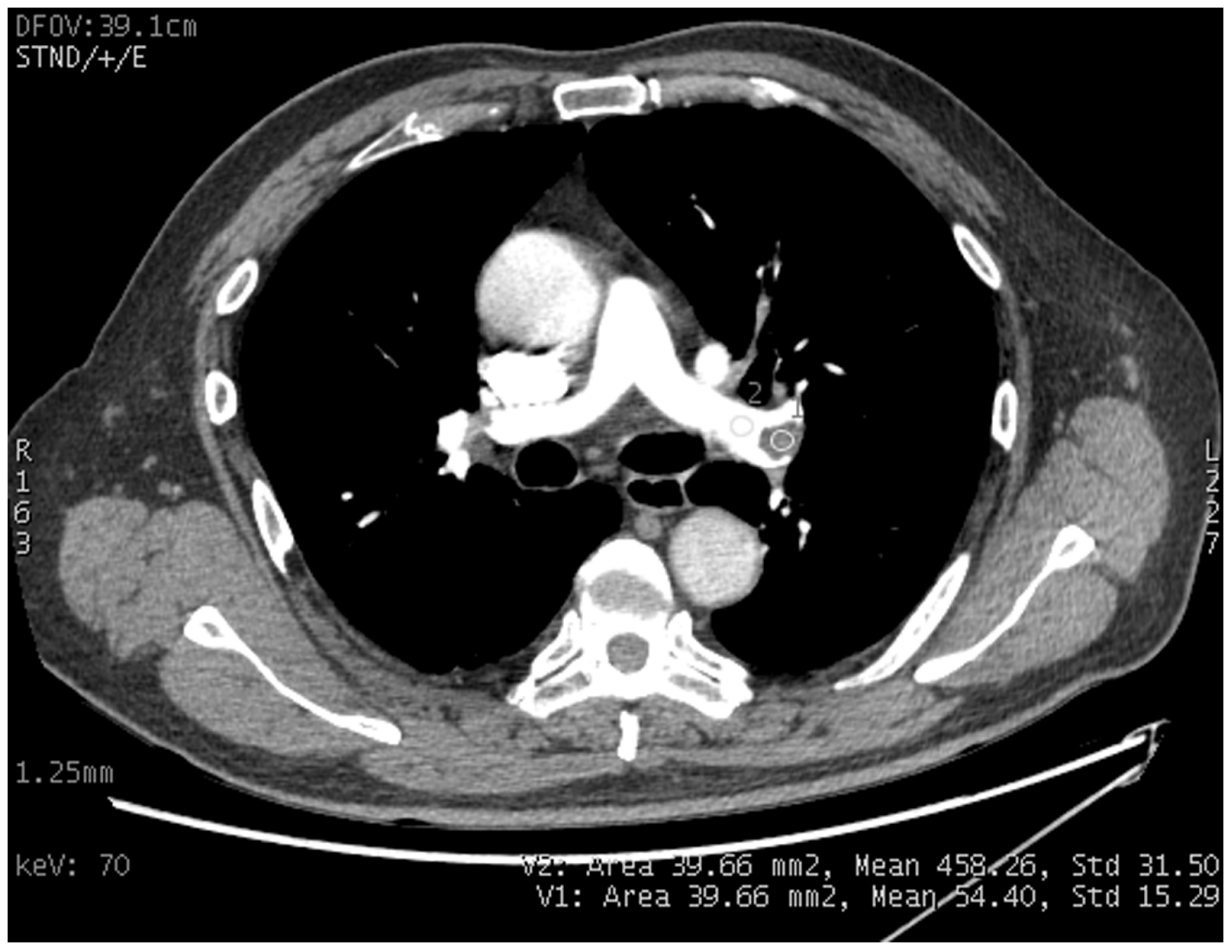
Enhanced axial image at pulmonary arterial phase from a patient with pulmonary embolism. The marked regions of interest are the endoluminal clot (diameter >5 mm) within the adjacent pulmonary artery.

The CT images were evaluated on an independent workstation (Advantage workstation 4.4: GE Healthcare). The GSI Viewer software package automatically calculated and displayed the CT attenuation values for the 101 sets of VMS images. Circular regions of interest (ROI) were placed on each endoluminal clot (diameter greater than 5 mm) and the adjacent area on the pulmonary artery carrying the clot to measure the mean CT values of both (Figure 1). The ROI sizes ranged from 22 to 81 mm^2^, based on the clot size. Each ROI was drawn to include at least two thirds of the object on each image series. A total of 58 clots were measured.

Contrast-to-noise ratio was defined according to the formula: CNR = (ROI_2_-ROI_1_)/SD_2_, where ROI_1_ denotes the CT value of the clot, ROI_2_ denotes the CT value of the pulmonary artery of the same slice, and SD_2_ denotes the standard deviation of pulmonary artery CT values. For endoluminal clot detection, the pulmonary artery carrying the clot was used as the contrast material. Background noise was determined as the standard deviation of air measured presternally in front of the patient. The CNR measurements were obtained from 101 sets of monochromatic images for the objective evaluation of CTPA. The GSI Viewer software package automatically calculated and displayed the CNR values for the 101 sets of images. A pre-evaluation was performed from the CNR plot to select 7 sets for subjective image quality assessment at the following energy levels: 50, 55, 60, 65, 70, 75 and 80 keV.

For subjective assessment, two radiologists with at least ten years of experience in thoracic imaging scored the axial monochromatic image series at the 7 energy levels for each of the following attributes: overall CTPA image quality, certainty of clot diagnosis, clot size and boundary estimation with the same window width, window level, and FOV. The subjective assessment was graded on a 5-point scale (5, excellent; 4, good; 3, adequate; 2, suboptimal; 1, unacceptable or poor).

### Statistical Analysis

All data were analyzed by using dedicated statistical software (SPSS for Windows, version 11.5). A value of p<0.05 was considered statistically significant.

Image noise values, contrast-to-noise ratios, clots diameters and subjective image quality were presented as mean ± standard deviation (SD). A paired T-test was performed on image noise, contrast-to-noise and clot diameter obtained from the different monochromatic image sets. Subjective image quality for sCTPA was tested using Wilcoxon rank sum test.

## Results

### Optimal Monochromatic Level for Image Noise

We first measured the mean image noise as function of monochromatic energy level (Table 1). The noise on the VMS images differed on the basis of the x-ray energy, with the minimum value observed at 65 keV. As the energy levels diverged from 65 keV, the noise of the CTPA increased. Compared with the 65 keV VMS images, the background image noise increases were 65.5% (50 keV), 33.8% (55 keV), 20.63% (60 keV), 4.4% (70 keV), 28.1% (75 keV), and 55.1% (80 keV) for the monochromatic images. The mean noise of the CTPA on the 65 keV VMS images was statistically lower than all other energy levels (p<0.01), with the exception of 70 keV where no difference was observed (p>0.05).

**Table 1:** Quantitative noise measurements as function of energy level.

Energy level (keV)	50	55	60	65	70	75	80
CNR	13.16±2.31	10.64±2.04	9.59±1.75	7.95±1.30	8.30±1.29	10.18±1.53	12.33±1.67

### Optimal Monochromatic Level for CNR

We next measured the mean CNR for clots as function of the monochromatic energy level (Table 2). CNR for the clot differed on the basis of the x-ray energy. The maximum CNR value was seen at 50 keV, which was statistically higher than those at all the other energy levels (p<0.01). The second highest CNR was obtained on the 65 keV VMS images with a value 9% lower than the maximum. The CNR at 65 keV was higher than that at 55, 75, and 80 keV (p<0.05). However, there was no difference in the mean CNR between the 65 keV and the 60 and 70 keV images (p>0.05).

**Table 2:** Quantitative CNR Measurements as function of energy level.

Energy level (keV)	50	55	60	65	70	75	80
CNR	20.03±4.57	17.25±3.61	18.1±4.22	18.31±3.88	18.19±4.08	16.12±3.12	12.37±3.96

### Clot Measurement

We then measured the mean clot diameter for 52 clots (diameter >5 mm) as function of the monochromatic energy level (Table 3). The mean clot diameters on the VMS images differed on the basis of the x-ray energy, and were decreased with the decrease of energy (keV) levels. There was difference in the mean clot diameters among the seven image sets (p<0.01). A total of 12 clots (small or threadlike embolus) disappeared or were significantly reduced in the low keV level images (50–55 keV).

**Table 3:** Quantitative clot diameter as function of energy level.

Energy level (keV)	50	55	60	65	70	75	80
Clot diameter (mm)	5.43±1.83	5.85±1.65	6.30±1.78	6.59±1.77	7.01±1.72	7.32±1.70	7.66±1.71

### Optimal Monochromatic Level for the Subjective Image Quality Assessment

**Figure 2:**
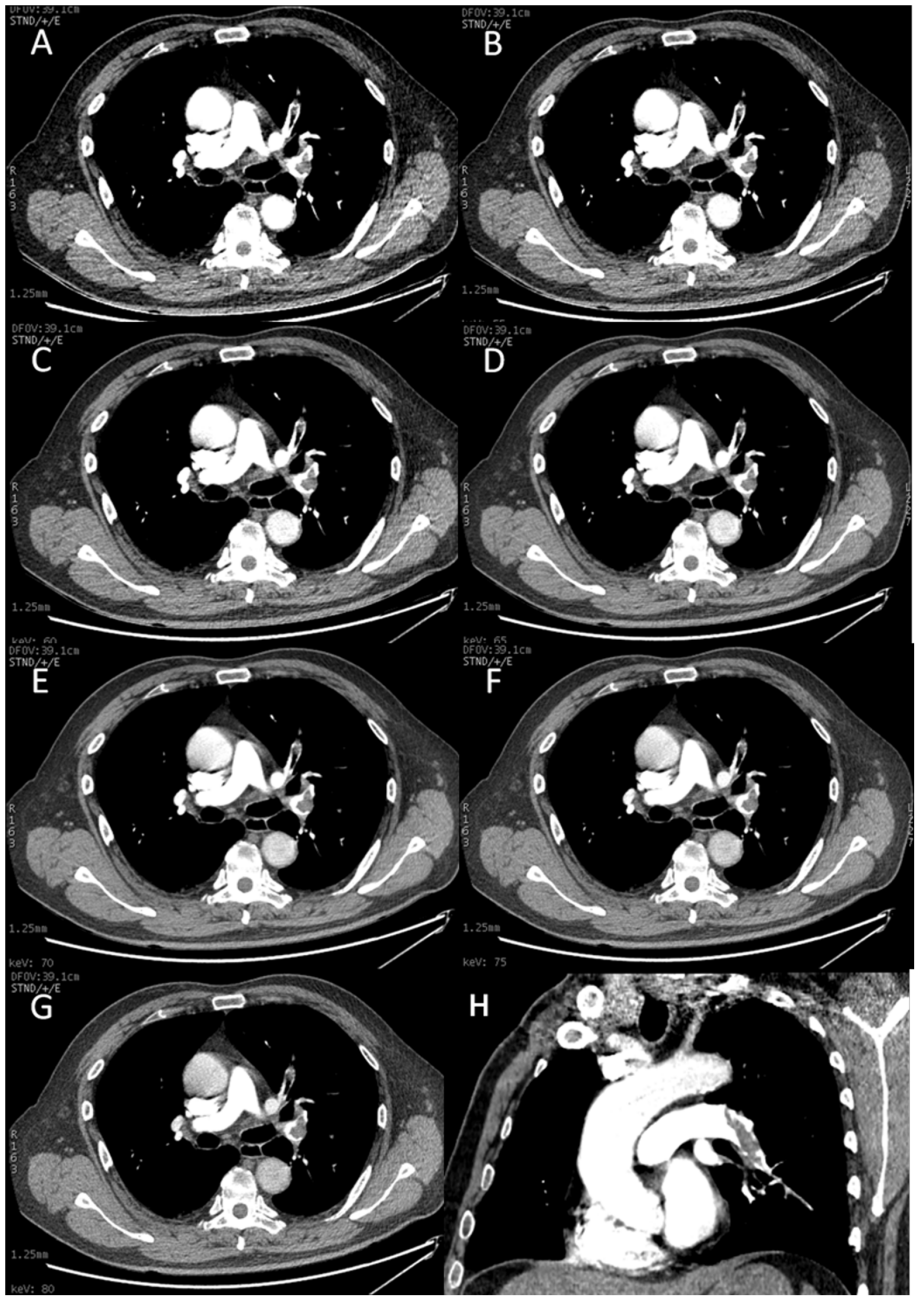
Comparison of subjective imaging scores. A, 50 keV; B, 55 keV; C, 60 keV; D, 65 keV; E, 70 keV; F, 75 keV; G, 80 keV. The 65 keV monochromatic image shows less image noise than 50, 55, 60 keV and high conspicuity when compared with the 75 and 80 KeV images. However, there was no significant difference in the subjective scores of the CTPA between the 70 and 65 keV images. H, oblique coronal CTPA image shows the endoluminal clot within the left inferior pulmonary artery.

Significant statistical difference was noted between subjective scores in the seven image sets (Table 4, Figure 2). The subjective image score for the 65 keV monochromatic CTPA was higher than the other image sets (p<0.05), except that at 70 keV (p>0.05).

**Table 4:** Subjective scores for image-quality assessment of VMS as function of energy level.

Energy level (keV)	50	55	60	65	70	75	80
Subjective scores	2.85±0.55	3.25±0.45	3.72±0.44	4.61±0.34	4.45±0.18	3.79±0.21	3.51±0.27

## Discussion

Computed tomographic spectral imaging enabled precise registration of data sets for the creation of accurate material decomposition images (e.g. water- and iodine-based material decomposition images), which arrive at quantitative density measurement. Virtual monochromatic spectral (VMS) images at energy levels ranging from 40 to 140 keV optimized angiographic imaging throughout the full 50 cm FOV.

As compared with widely used polychromatic imaging, VMS images reconstructed with more accurate beam-hardening correction provide improved linearity of CT attenuation, and improved CNR. We measured 101 VMS image sets obtained in the range of 40 to 140 keV. This provided an opportunity to select the optimal energy level for detecting and evaluating endoluminal clots. Theoretically, the lowest energy levels would yield the ‘‘brightest’’ iodine and the greatest image contrast, and low kilovolt (peak) (kVp) and dual-energy CT can exploit the K-edge phenomenon to improve the contrast for vessels when contrast agent is used. However, the highest CNR does not normally occur at the lowest energy level as image noise usually increases as photon energy decreases. Finding the highest CNR proved to be advantageous in angiographic imaging [18]–[20]. On the other hand, detecting and evaluating non-enhancing endoluminal clots in enhancing vessels requires more than just optimizing CNR. Other variables including image noise, overall image quality, clot conspicuity, clot size and boundary estimation are also important. Excessive image noise or contrast concentration in the vessel at low energy levels may influence the display of emboli, making it difficult to determine the boundary and size of clots, especially small clots. In our study, a total of 12 smaller or threadlike emboli disappeared (or their conspicuity was significantly reduced) with low photon energy levels (50–55 keV). This phenomenon was most likely due to the partial volume effect by high vascular enhancement. Thus, CNR and image noise must be balanced to optimize the detection of pulmonary embolism, just as we use specific window and level settings for particular indications.

Although our results indicate that highest CNR for a clot occurred at low energy (50 keV), the lowest image noise and highest subjective image score was obtained in VMS images at approximately 65 keV. As the photon energy diverged from 65 keV, the noise of the CTPA increased. Compared with the 65 keV VMS images, the background image noise level was increased at all other energy levels (4.4–65.5% increase). The subjective image quality score followed a similar pattern with the highest image quality score occuring at approximately 65 keV. However, there was no statistical difference in the image noise, CNR and quality score between the 70 and 65 keV (p>0.05), even though numerically 65 keV had slightly higher mean values than 70 keV. In addition, CNR for both the 65 keV and 70 keV VMS images were only 9% lower than the highest value at 50 keV, providing adequate contrast-to-noise ratio for the evaluation of pulmonary embolism. Our results are consistent with the results of the previous phantom study using a vascular phantom with idealized anatomic environment, where Matsumoto *et al.* found virtual monochromatic spectral (VMS) images at approximately 70 keV yielded lower image noise and higher CNR than did 120 kVp CT for a given radiation dose [21].

This retrospective study does have several limitations. First, we are concerned about the appropriate contrast of emboli and vascular enhancement, so our study group was limited to PE patients with a large clot size. Second, a comparison of the noise or CNR between the VMS images and conventional pure 120 kVp images would be desirable. However, both of the simulated weighted-average 120 kVp images and 80 kVp images could not be generated from the data sets of the fast tube voltage switching dual-energy CT scanning mode. Additional acquisition of pure 120 kVp (or pure 80 kVp) images at different times will also result in an increase of radiation exposure of the participating patients. Therefore, we did not compare the VMS images with simulated 120 kVp images. Third, image analysis was conducted by consensus between two readers and did not include the evaluation of inter-observer variability in the analysis of peripheral arteries. Because this study was aimed at analyzing differences in objective image quality, we favored the consensus analysis. Lastly, we had no gold standard for the measurement of the clot diameter. So we did not know which energy level gave the most accurate values for the clots, and further *in vitro* experiments are warranted.

In conclusion, virtual monochromatic spectral images at approximately 65–70 keV in spectral CT pulmonary angiography yielded the lowest image noise, high contrast-to-noise ratios and highest diagnostic confidence for detecting, diagnosing and evaluating pulmonary embolism.
